# Why human connection is the true metric of research success

**DOI:** 10.1002/2211-5463.70238

**Published:** 2026-04-09

**Authors:** Timothy Lin Yun Tan, Mattias Wei Ren Kon, Xavier Coumoul, Fun Man Fung

**Affiliations:** ^1^ Faculty of Science National University of Singapore Singapore; ^2^ Yong Loo Lin School of Medicine National University of Singapore Singapore; ^3^ Université Paris Cité INSERM, Health & Functional Exposomics – HealthFex France; ^4^ School of Chemistry University College Dublin Ireland; ^5^ UCD Geary Institute for Public Policy University College Dublin Ireland

**Keywords:** Academic Mentorship, Doctoral Education, Early Career Researchers, Higher Education Policy, Research Culture, Researcher Well‐being, students as partners

## Abstract

Postdoctoral supervision and research leadership are crucial yet underexamined dimensions of academic work. In this perspectives piece, we reflect on mentorship across Singapore and France and situate it within output‐driven research ecosystems that risk reducing groups to production units. Using a narrative and workshop‐based approach, including a mentorship workshop at the FEBS‐IUBMB ENABLE 2024 conference in Singapore, we explore how mentees define good mentorship. Participants consistently prioritised everyday human interactions over traditional metrics, highlighting empathy, trust, humility, availability, and clear communication. Integrating these insights with educational literature, we illustrate that humane, ethically grounded mentorship is essential for research integrity, well‐being and sustainable scientific capacity.

## Graphical abstract

Human‐centred mentorship can be shaped by mentor attributes, actions, intrinsic drive and career ambition. Drawing on reflections across Singapore and France, as well as workshop insights from FEBS‐IUBMB ENABLE 2024, this article shows that human‐centred mentorship creates the conditions for sustainable growth, well‐being and retention in research environments.

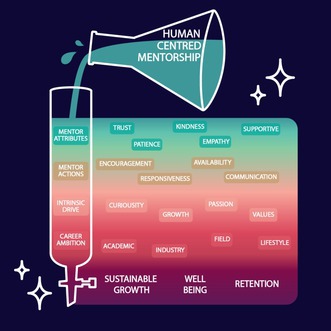

**Abbreviations**
A*STAR: Agency for Science, Technology and Research
ENABLE: European Academy for Biomedical Science
FEBS: Federation of European Biochemical Societies

## Introduction

The authors chanced upon an article published by Rostalski *et al*., [1], on the topic ‘Why emerging leaders should focus on being good postdoctoral supervisors’. In this paper, Rostalski *et al*., use the landscape of postdoctoral supervision in their institution to discuss strategies in which more effective student–supervisor relationships can be forged, enabling postdoctoral researchers to be more prepared for their future career where leadership duties may be involved. In alignment with the messaging from Rostalski *et al*., and FEBS Open Bio's objective of supporting the research community [2], the authors from Singapore and France wish to take a step back to examine the broader notion of mentorship and the characteristics defining an effective mentorship, through their own lived experiences and perspectives.

## What we see around us: leadership in the 21st century research group

Just like all potential readers of this article, everyone's journey in research began on our first day as part of a research group. The individuals we meet there, from fellow undergraduate students to PhDs, employed research assistants to postdoctoral fellows, and of course, the lab PI. All of these individuals do not solely play their roles in making the research group function, but each of them has the potential to be a supervisor to someone more junior than them, though this responsibility often falls onto the shoulders of the PI. When we have queries, we seek their input for clarity. When we have doubts about our career goals, we confide in them for life advice. Every supervisor we encountered made a difference in our lives in one way or another.

However, a good researcher, supervisor or thesis advisor may not necessarily make a good leader, and by extension, a good mentor [3, 4, 5]. When we discuss the research group as an outcome‐oriented team, the output takes the lead. But leadership and mentorship are not merely outcome‐oriented, it involves both a professional and a personal relationship. Kirk *et al*., [6, 7] from Australia suggested in a 2017 study that good mentors play a crucial role in charting a junior researcher's academic journey and career aspirations, across all fields of academia. Contemporary European scholarship similarly frames mentorship as a developmental, reciprocal relationship that supports career progression, professional identity formation and psychosocial well‐being [8, 9, 10, 11]. Across European and international higher education and workplace settings, high‐quality mentoring is associated with improved performance, promotion, salary progression, expanded professional networks and enhanced sense of belonging, confidence and well‐being [8, 10, 12, 13]. Such relationships are typically characterised by mutual respect, clear and negotiated expectations, regular and open communication, and deliberate attention to power dynamics, equity and inclusion [14, 15, 16]. Projects such as GAELIC MENTORS (hosted at University College Dublin School of Chemistry) operationalise these findings by utilising trained senior peer mentors to bridge the gap between academic performance and social‐psychological well‐being.

Additionally, the abovementioned characteristics defining good mentorship are known to perpetuate as researchers tend to adopt the approaches in which they were ‘raised’ on [17]. This question has been well‐studied especially in fields such as medicine and nursing, where a hallmark paper by Taylor [18] in 1992 on nursing academicians found that well‐established mentor‐protege relationships contributed towards improved professionalism, integrity and other core competencies. As such, mentorship under the broader premise of research supervision is understandably a key variable influencing a junior researcher.

## What is the issue: churning out papers, but also superficial relationships

The research journey is one plagued with celebrated milestones, yet also with its enduring challenges. As student progress from year to year in pursuit of their degrees, they envision becoming one step closer to their dream job, closer to achieving their career goals. One of the well‐established measures in guiding junior researchers is publication, through scales such as journal impact factor or the Hirsch, or h‐index [19]. Studies indicate that these impact measures play significant roles in determining a junior researcher's subsequent career progression in academia [20, 21, 22].

However, the path to academic success is rarely straightforward. Beyond the contentions regarding an over‐reliance on journal reputation and h‐index [22, 23], these measures have fostered a ‘publish or perish’ culture in various institutions, turning collegiality into rivalry and mentorship into a transactional investment [24, 25, 26, 27, 28]. Through superficial interactions compounded by mismatched expectations, these ‘mentors’ can hinder the professional development of junior researchers [29, 30]. Where a human connection marked by care, concern, and camaraderie should exist, there are often only infrequent meetings, unread messages, and awkward, superficial exchanges.

This competitive environment has contributed to an epidemic of stress among junior researchers, leading to anxiety, depression, and burnout [31, 32]. Unsurprisingly, a study by Evans *et al*. [33] in 2018 found that a majority of the *n* = 2279 surveyed individuals who experienced anxiety (49%) and depression (50%) thought that their PI failed to provide them with ample support. Worse, the Nature PhD Survey 2019 found that 21% of surveyed PhD students experienced some form of discrimination or harassment by their supervisors in the workplace [34]. These findings reinforce the notion that a genuine, supportive mentor–mentee relationship is critical [35].

It is here that Singapore presents a compelling context in which to explore these mentorship dynamics. Despite its geographic size, the nation has established itself as a globally recognised research hub, achieved through the deliberate cultivation of a robust ecosystem and substantial economic investment. Platforms such as the Singapore Science Conferences (SSC), which convene thousands of scholars worldwide including over 1200 delegates in 2025 alone, exemplify this commitment to academic exchange. In many ways, it mirrors numerous European research systems shaped by excellence initiatives and competitive funding schemes. Yet, such structures have intensified pressures around research visibility and output, often at the expense of the relational aspects of academic work [36]. The very success of this infrastructure thus raises an important question: does the intense focus on research visibility and output inadvertently overshadow the role of developmental coaching?

To answer this, one must examine how the local academic culture shapes perceptions of mentorship. It remains to be seen whether the value of mentorship is recognised equally across hierarchical lines, or if it is viewed as secondary to tangible production. Furthermore, while various professional development policies exist, it is unclear if current incentives are sufficient to nurture quality mentorship or if significant gaps remain regarding equal access across different demographic groups and institutions.

From an observational perspective, there appears to be a systemic inclination—among institutions and academics in Singapore and many European settings—to prioritise objective, quantifiable measures like publication output over subjective measures such as student feedback. Also, the EU's growing obsession with ‘impact’ in funded projects has become both a guiding principle and a source of distortion, particularly when coupled with the intense pressure placed on postdoctoral management. While the rationale is understandable (public investment should translate into societal, economic or policy benefits), the practical consequences are more troubling. Postdocs who get involved in EU projects (a great international experience for sure) are no longer seen primarily as early career scientists in a formative phase, but as quasi‐project managers, deliverable machines and living proof of impact generation. They are expected to publish fast, coordinate stakeholders, contribute to dissemination, supervise students (PhD, masters…), manage work packages and embody ‘capacity building’, often within short, precarious contracts.

This combination creates a structural tension: The system demands bold, innovative science, yet organises labour around hyperaccountability and constant performance. In the end, insufficient project oversight by the PIs often shifts excessive responsibility onto postdoctoral researchers who are expected to make strategic decisions, absorb uncertainty and ensure delivery without the authority, stability or institutional support that such responsibilities would normally require. We do not imply that mentorship is intentionally neglected in Singapore, Europe or similar high‐performance environments. Rather, the dominance of output metrics can crowd out necessary discourse regarding the relational aspects of research. There is, therefore, a growing opportunity to complement Singapore's scholarly strengths by attending more intentionality to human relationships. This expansion of focus does not require a reduction in rigour or ambition; rather, it suggests that supporting the people who carry out research is as vital as the pursuit of knowledge itself.

France offers an instructive counterpoint to these dynamics. The French academic system is often perceived as highly hierarchical, yet it also incorporates structural safeguards that implicitly acknowledge the limits of mentoring capacity. For instance, by law, a professor may formally supervise only a small number of PhD students at any given time, typically no more than three as primary supervisor, reflecting a simple but often overlooked reality: effective mentorship requires time, presence and cognitive availability. Moreover, cosupervision is allowed to ensure a better training of PhD students. Beyond individual supervision, doctoral training in France is largely organised through doctoral schools (e.g. https://ed563.u‐paris.fr), which provide structured oversight, establish mentoring committees and conduct regular progress evaluations involving multiple senior academics. Universities and research organisations such as Inserm or CNRS have also developed programs aimed at improving laboratory climate and mentoring quality, including supervisor training, mediation and mechanisms to address conflicts or power imbalances.

While these frameworks do not guarantee exemplary mentorship, they nevertheless signal an institutional recognition that supervision is not infinitely scalable. From this perspective, the French model highlights a central tension: When even regulated systems struggle to ensure high‐quality mentorship as research teams expand, the expectation placed on academics, already absorbed by grant acquisition, expert duties and administrative responsibilities, to supervise ever‐growing numbers of PhD students and postdoctoral researchers becomes increasingly unsustainable.

In this piece, the authors aim to characterise the nuances underlying such a good mentor–mentee relationship, while also tapping on the personal experiences of the authorship team. Furthermore, external perspectives from other students and supervisors from countries all over the world are reflected upon and integrated with that of the authors to tell a story about what it means to be human in the academic environment.

## Our story: a team of diverse backgrounds

The authorship team comprises Timothy—a current PhD student, Mattias—a current medical student, Xavier—a biochemistry and toxicology University professor and FEBS member, and Fun Man—a chemical education researcher and PI of the Senpai Learn research group. Timothy joined the group when he was an undergraduate student at the National University of Singapore (NUS) reading a module taught by Fun Man, and was interested to explore chemical education further as one of his research interests. Meanwhile, Mattias joined the group when he was a high school student at a specialised math & science‐focused high school. Through the NUS High School SCIENTIA programme, Mattias and 2 of his peers joined the Senpai Learn group to gain pre‐university exposure to academia and formal research. Xavier and Fun Man have worked together on numerous occasions as part of France‐Singapore research partnerships, having previously met at past FEBS Congress [37, 38].

Since 2022, when Timothy and Mattias joined Senpai Learn to work on the ChemPOV project, developing a digital, multiplayer board game for organic chemistry learning, the team has achieved numerous milestones together [39]. Timothy represented the team at the 9th Network of Inter‐Asian Chemistry Educators Conference (9NICE) 2023 in Kuching, Malaysia, while Mattias and his peers from NUS High School presented their work at the ACS Spring Meeting 2023 in Indianapolis, USA, IUPAC World Chemistry Congress 2023 in Den Haag, Netherlands, and the 27th IUPAC International Conference on Chemistry Education (ICCE) 2024 in Pattaya, Thailand [40].

The ChemPOV team continued working together even after Timothy graduated with his Bachelor's, Mattias graduated from high school, and Fun Man moved his research group from Singapore over to Dublin, Ireland with University College Dublin. The team published two studies featuring our work on ChemPOV, which has been trialled on both high school and undergraduate level students [41, 42]. Having travelled to various parts of the world together for research, the ChemPOV team stayed connected even though our paths gradually diverged.

This collaboration naturally intersected with the work of Xavier, a professor of biochemistry and toxicology at Université Paris Cité, who, through his teaching activities, has always been interested in educational innovations, both digital and non‐digital. It was in this context that he visited the National University of Singapore in 2015 and met Fun Man, who was particularly interested in innovative teaching technologies developed for chemistry education. Their interdisciplinary collaboration on the scientific level and complementary collaboration on the educational level led them to develop digital tools to facilitate learning and the development of critical thinking among students at both universities. They have both been involved in the activities of the education committee of the FEBS, either as committee members or as guests of its committees, participating in FEBS conferences. Their collaboration continues, especially since Fun Man has moved closer to France by establishing a permanent presence in Ireland, and this discussion gains new urgency within the Irish Higher Education landscape. Our emphasis on relational mentorship aligns with the HEA EDI Enhancement Fund 2025 (https://hea.ie/2025/08/29/equality‐diversity‐and‐inclusion‐enhancement‐fund‐2025‐announcement/), specifically within projects like GAELIC MENTORS. This project recognises that sustainable scientific capacity is built on inclusive, equitable learning environments that prioritise the human element of chemistry education.

## Our journey: charting the path of mentorship

The opportunity for us to truly explore and delve deeper into what mentorship meant to us arrived in the form of an invitation by the FEBS‐IUBMB ENABLE Conference organising team for the 2024 congress held at the Lee Kong Chian School of Medicine, Nanyang Technological University, Singapore [43]. This was the first time this conference was held in Asia, or outside Europe for that matter. Fun Man was asked to deliver a workshop exploring the topic of mentorship while he was just beginning to settle down into his new position in Ireland. Thus, he entrusted the organisation and running of the workshop to both Timothy and Mattias as he joined the session virtually and empowered them to add in their own perspectives to the workshop with the understanding that students have a lot—if not more—to say about what makes a good mentor than a supervisor would.

**Fig. 1:**
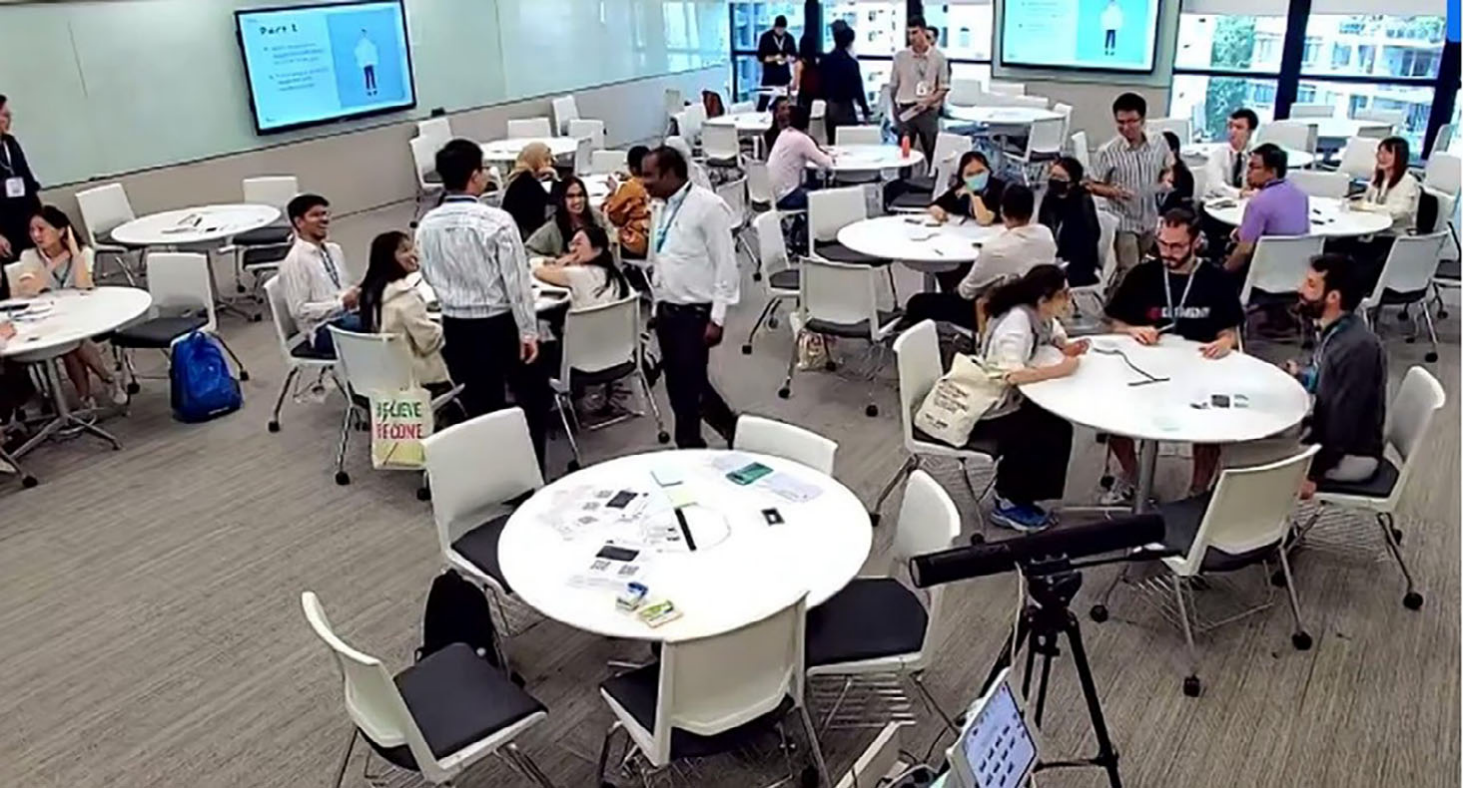
FEBS‐IUBMB‐ENABLE symposium workshop in‐progress.

With that, the session took place during the symposium alongside various parallel symposia and workshops. We attained a high level of attendance, with 27 attendees out of the 30 total seats for participants. The participants came from 11 different countries and were at varying stages of their academic journey or career progression, with over half of them being doctoral students or postdoctoral researchers (Fig. 1).

While exploring the nuances of mentorship, we also wanted participants to leave feeling inspired and empowered. To support this, the workshop structure was grounded in experiential and collaborative learning principles. Drawing on inspiration from Hokanson [44] and Li *et al*., [45] the design prioritised active participation, peer dialogue and reflection rather than didactic instruction. In parallel, we utilised a reflective journalling approach to promote self‐reflection and invite participants to articulate their own understanding of mentor–mentee relationships. Building on work from Machost and others [46, 47], we aimed to use this guided reflection to deepen self‐awareness, empathy and interpersonal competence in mentoring and educational contexts [48, 49]. Such techniques have been widely adopted across educational sectors to foster critical and intentional thinking [50, 51]. In particular, medical and nursing education have used these approaches to instil clinical judgement and teaching skills [52, 53]. Together, these principles create a social, participatory environment in which participants can strengthen communication, confidence and higher‐order thinking skills around mentorship.

## Our findings: how mentorship is experienced and evaluated

During the workshop, the lead facilitators—Timothy and Mattias, spoke about their personal experiences with their own academic journeys, while Fun Man closed the introductory segment with an address on how mentorship shaped his career from being an educator passionate about teaching, to a researcher passionate about further improving his teaching. Thereafter, the facilitators encouraged participants to reflect on their thoughts regarding 3 main questions about mentorship: (a) What does it mean to be a good mentor, (b) what does it take to be a good mentor and (c) how do you think you could have been a better mentor, or how could someone have been a better mentor to you? These were noted down onto post‐its by participants and stuck onto a board for each discussion group. Each group comprised between 3 to 5 participants.

**Fig. 2:**
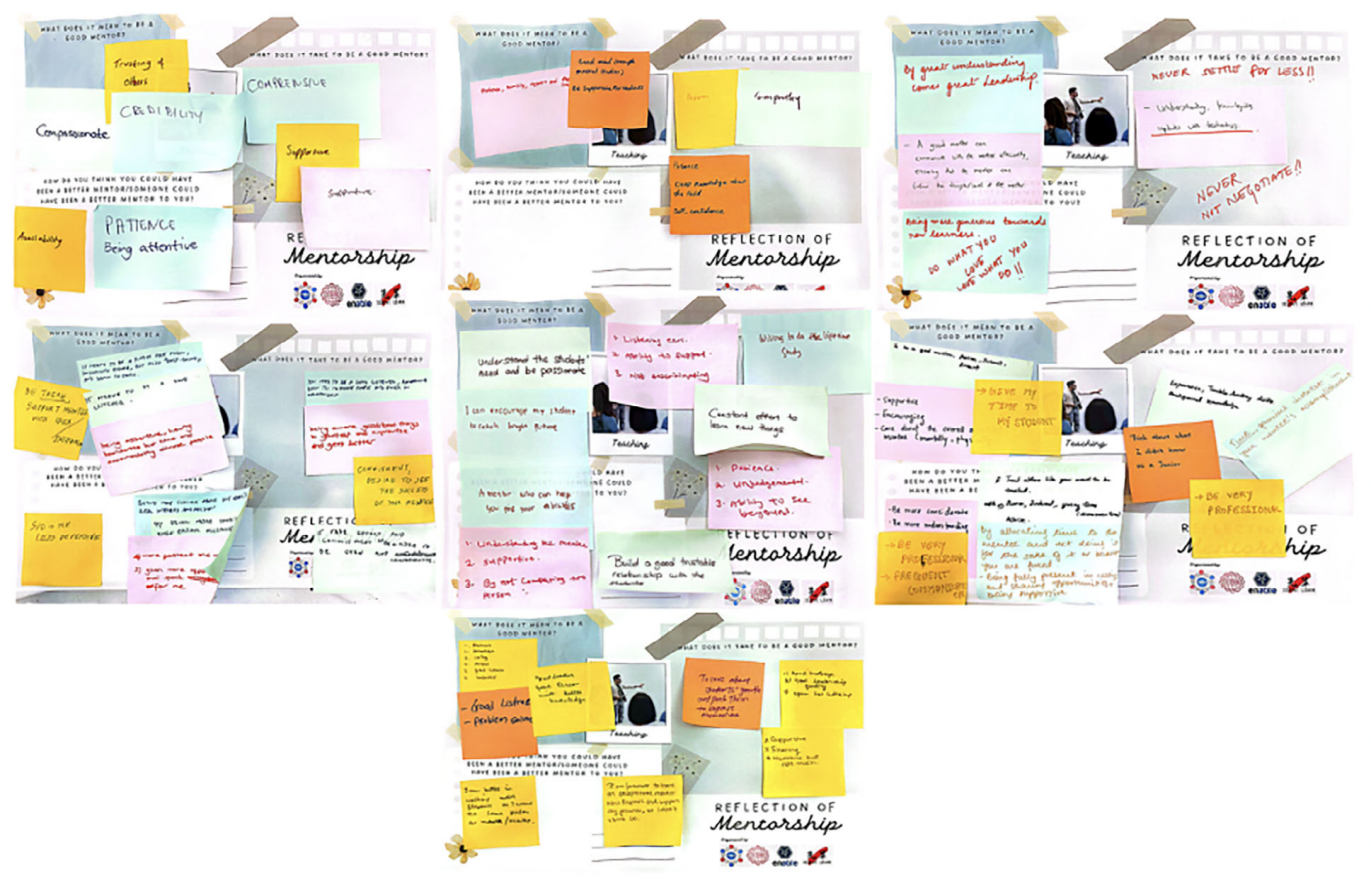
Participants' responses to the 3 posed questions on mentorship, written on post‐its and pasted onto a board.

Through the course of the workshop, a consistent theme emerged: mentorship is often judged based on everyday human interactions rather than results (Fig. 2). When asked about what defined a good mentor, participants rarely mentioned tangible outcomes such as grants and publications. Instead, there was an undeniable emphasis on connection between mentor and mentee—how seen, heard, or supported they felt. In terms of attributes, interpersonal qualities such as trust, compassion, patience, humility, respect and kindness appeared across all groups, regardless of disciplinary background or career stage. Other behavioural markers of effective mentorship that were consistently brought up included availability, communication and care/consideration. This suggests that mentorship is grounded more strongly in human interaction than traditional output‐driven metrics of success.

## Our reflections: why mentorship should be about being human

Even as our team is situated at different stages of our academic journeys, we, too, have experienced being recipients of impactful mentorship and acting as mentors learning to navigate the balance between productivity and humanity. Such experiences have shaped our view that good mentorship is as much relational as it is instructional. From our perspective, one of the most striking observations is how mentorship is often evaluated not through outcomes, but through emotional lenses. When people speak about their mentors, they rarely begin with publication counts or grant totals. Instead, they talk about how a mentor responded when they were struggling, whether they felt comfortable asking questions or whether their mentor took the time to listen when things felt uncertain. Such emotional aspects, while harder to quantify, often carry far greater weight in shaping a mentee's confidence, sense of belonging and willingness to persist in academia [54].

At the same time, many of these shortcomings in mentorship we observed were often not born of malice or neglect, but of misaligned expectations and assumptions. Senior researchers are frequently stretched thin, having to balance administrative responsibilities, funding, their own research and possibly teaching obligations, all while being expected to support junior mentees. On the flip side, junior researchers may hesitate to articulate their needs due to power dynamics or fear of being seen as underperforming. In such contexts, even well‐intentioned mentorships can become (unintentionally) fragmented and distant.

It is our view, then, that while a certain degree of technical guidance and expertise is necessary, a purely instructional relationship is often insufficient in the fostering of a mentee. Effective mentorship also requires trust, consideration and a willingness to engage mentees as their own person, that is being human.

**Fig. 3:**
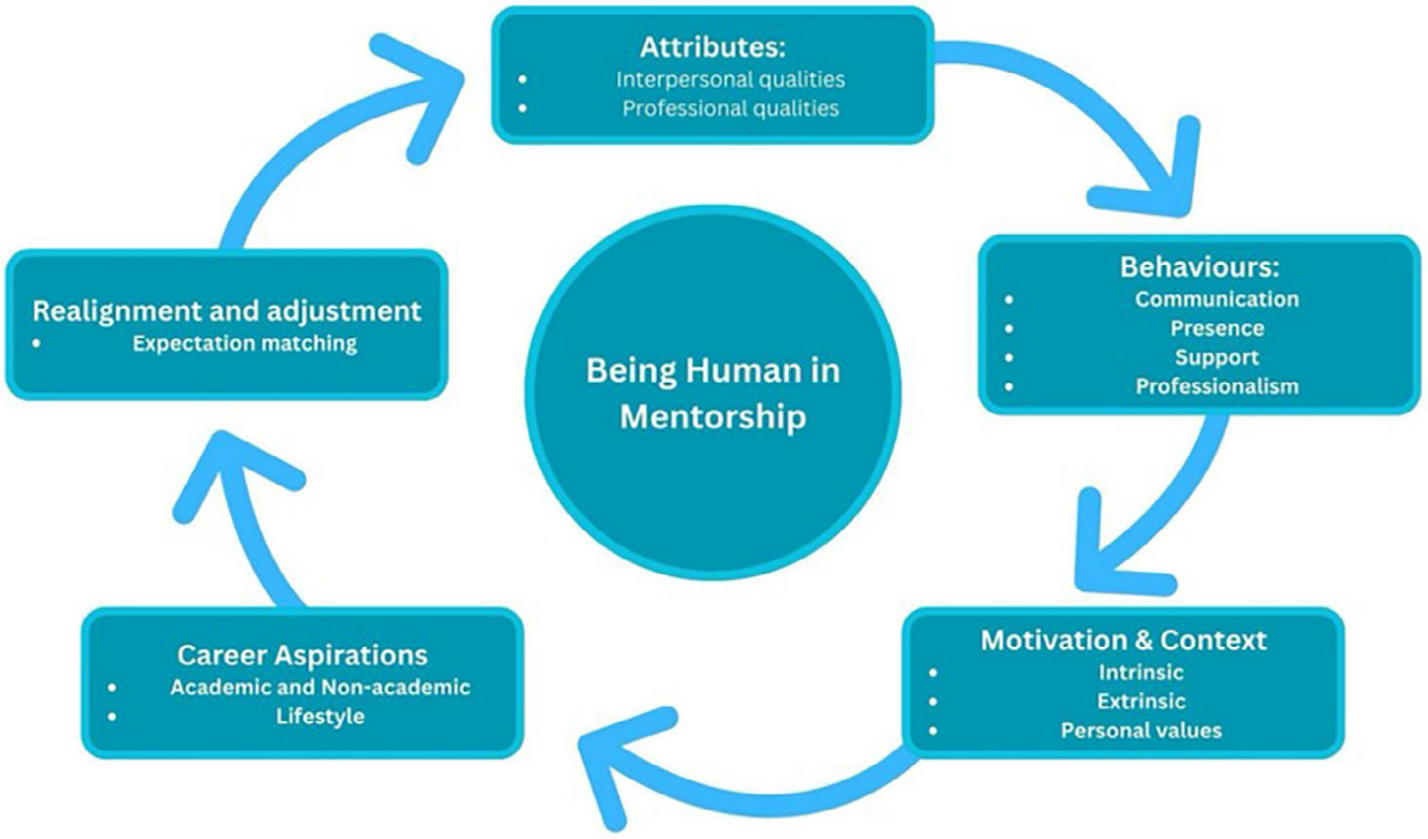
A schematic of what it means to being Human in Mentorship.

At its core, a human‐centred mentorship is inseparable from ethics and scientific integrity, not as abstract principles, but as daily practices at the bench and beside the bench (e.g. writing an article). Being human as a mentor means encouraging openness: openness to other disciplines, which invites postdocs (and beyond) to step outside their comfort zone, renew their curiosity and sometimes even reinvent their scientific identity by exploring new fields (Fig. 3). It also means providing very concrete forms of support, such as a desk, protected time, a bench for experiments, all while codefining achievable objectives with the postdoc rather than imposing them unilaterally. Additionally, extra consideration needs to be given in the provision of such spaces; uneven distribution (even when done unintentionally) of resources can very quickly become pain points due to comparison, leading to members feeling undervalued and neglected. Regular, structured discussions around milestones are not only tools for productivity, but moments of dialogue that acknowledge uncertainty, doubt and learning as integral parts of research.

A human approach to mentorship also actively values multiculturalism and peer exchange, fostering discussions among postdocs with diverse backgrounds and trajectories. Finally, collaboration must be organised with care: Joint projects can be powerful spaces for collective growth, provided that mentors clearly define rules for authorship and recognition from the outset, avoiding competition that erodes trust. In this sense, mentorship is not merely about supervising research; it is about creating a fair, open and humane ecosystem in which integrity, collaboration and personal development can genuinely coexist.

In Singapore, there already exists substantial infrastructure and several programmes designed to incentivise effective mentorship. For example, the STAR Mentor Award is part of A*STAR's broader initiative to recognise and inspire excellent mentors.

However, we believe that despite these external incentives, the current system would benefit from additional initiatives that focus on what the core principles of effective mentorship, cultivating said principles and protecting dedicated time for mentors to develop their mentoring skills. However, we do recognise that further research is needed in this domain of human‐centred mentorships, and how to effectively manage them. Such a challenge aligns with the goals of organisations such as A*STAR Institute of Human Development and Potential (IHDP), which are dedicated to improving the health and well‐being of our population.

## In a nutshell: relationships matter most, above everything else

When we graduate a PhD student, we graduate a scientist, not a thesis. Relationships matter most because mentoring, and ultimately leadership, is fundamentally a developmental rather than a transactional relationship. What transforms mentees is trust, empathy and compassion within the mentor–mentee bond, more so than technical expertise and advice. The workshop further illustrated impactful mentoring as one that offers support, advocacy and genuine care, which enables researchers to persist in academia.

## Conflict of interest

The authors declare no conflict of interest.

## Author contributions

T.L.Y.T., M.W.R.K. contributed to conceptualising, drafting and editing of the manuscript. X.C. contributed to reviewing and editing of the manuscript. F.M.F. contributed to conceptualising, reviewing and editing of the manuscript. All authors have read and approved the final version of the manuscript and agree to be accountable for its contents.

## Data Availability

The data that support the findings of our reflection are available on request from the corresponding author, F.M.F.

## References

[1] Rostalski H , Oudenaarden C , Tian M , Gopaul D , Kurilla A and Ersan PG (2025) Why emerging leaders should focus on being good postdoctoral supervisors. FEBS Lett 600, 3–9. 41204621 10.1002/1873-3468.70214

[2] Fuentes S and De la Rosa MA (2026) A step further in our commitment to support early career researchers (ECRs). FEBS Open Bio 16, 4–10. 10.1002/2211-5463.70187 PMC12767765 41489992

[3] Bird SJ (2001) Mentors, advisors and supervisors: their role in teaching responsible research conduct. Sci Eng Ethics 7, 455–468. 11697001 10.1007/s11948-001-0002-1

[4] Houser C , Lemmons K and Cahill A (2013) Role of the faculty Mentor in an undergraduate research experience. J Geosci Educ 61, 297–305.

[5] Rankin EA (1991) Mentor, mentee, mentoring: building career development relationships. Nursing Connections 4, 49–57. 1791870

[6] Kirk M and Lipscombe K (2019) When a postgraduate student becomes a novice researcher and a supervisor becomes a mentor: a journey of research identity development. Stud Teach Educ 15, 179–197.

[7] Gray M and Roy C (2005) Role of the Supervisor/Mentor. Doctoral Education in Nursing, 143–60.

[8] Blake H (2025) Workforce career development in public health, health education, and the health services: insights from 30 years of cross‐disciplinary national and international mentoring. Int J Environ Res Public Health 22, 729. 40427846 10.3390/ijerph22050729 PMC12111403

[9] Chen Y‐C and Chai C‐C (2023) Development of a two‐way mentorship scale focusing on next‐generation core competencies. Humanities and Social Sciences Communications 10, 629.

[10] Hryshchenko M , Artemchuk M , Zavhorodnya L , Tymoshenko Y and Purhani S (2025) The role of mentoring in the employee professional development and career growth. Sapienza Int J Interdiscip Stud 6, e25004.

[11] Sarabipour S , Hainer SJ , Arslan FN , de Win CM , Furlong E , Bielczyk N , Jadavji NM , Shah AP and Davla S (2022) Building and sustaining mentor interactions as a mentee. FEBS J 289, 1374–1384. 33818917 10.1111/febs.15823 PMC8490489

[12] Shier ML , Larsen‐Halikowski J and Gouthro S (2020) Interpersonal dynamics shaping positive mentee and mentor relationships. Child Adolesc Soc Work J 37, 497–509.

[13] Zaniewski AM and Reinhol D (2016) Increasing STEM success: a near‐peer mentoring program in the physical sciences. Int J STEM Educ 3, 14.

[14] Mullen CA and Klimaitis CC (2021) Defining mentoring: a literature review of issues, types, and applications. Ann N Y Acad Sci 1483, 19–35. 31309580 10.1111/nyas.14176

[15] Pfund C , Byars‐Winston A , Branchaw J , Hurtado S and Eagan K (2016) Defining attributes and metrics of effective research mentoring relationships. AIDS Behav 20, 238–248. 27062425 10.1007/s10461-016-1384-z PMC4995122

[16] Pfund C , Sancheznieto F , Byars‐Winston A , Zarate S , Black S , Birren B , Rogers J and Asai DJ (2022) Evaluation of a culturally responsive mentorship education program for the advisers of Howard Hughes Medical Institute Gilliam program graduate students. CBE‐Life Sci Educ 21, ar50. 35862583 10.1187/cbe.21-11-0321 PMC9582832

[17] Lee A (2007) Developing effective supervisors: concepts of research supervision. S Afr J High Educ 21, 680–693.

[18] Taylor LJ (1992) A survey of mentor relationships in academe. J Prof Nurs 8, 48–55. 1573116 10.1016/8755-7223(92)90117-h

[19] Chapman CA , Bicca‐Marques JC , Calvignac‐Spencer S , Fan P , Fashing PJ , Gogarten J , Guo S , Hemingway CA , Leendertz F , Li B *et al*. (2019) Games academics play and their consequences: how authorship, h ‐index and journal impact factors are shaping the future of academia. Proc R Soc Lond B Biol Sci 286, 20192047. 10.1098/rspb.2019.2047 PMC6939250 31797732

[20] Van Dijk D , Manor O and Carey LB (2014) Publication metrics and success on the academic job market. Curr Biol 24, R516–R517. 24892909 10.1016/j.cub.2014.04.039

[21] Tregellas JR , Smucny J , Rojas DC and Legget KT (2018) Predicting academic career outcomes by predoctoral publication record. PeerJ 6, e5707. 30310749 10.7717/peerj.5707 PMC6174868

[22] Tregoning J (2018) How will you judge me if not by impact factor? Nature 558, 345. 29921857 10.1038/d41586-018-05467-5

[23] Abbott A , Cyranoski D , Jones N , Maher B , Schiermeier Q and Van Noorden R (2010) Metrics: do metrics matter? Nature 465, 860–862. 20559361 10.1038/465860a

[24] Clapham P (2005) Publish or perish. Bioscience 55, 390–391.

[25] Kendal D , Lee KE , Soanes K and Threlfall CG (2022) The great publication race vs ‘abandon paper counting’: benchmarking ECR publication and co‐authorship rates over past 50 years to inform research evaluation. F1000Res 11, 95.

[26] Angell M (1986) Publish or perish: a proposal. Ann Intern Med 104, 261–262. 3946958 10.7326/0003-4819-104-2-261

[27] Abdollahi M , Gasparyan AY and Saeidnia S (2014) The urge to publish more and its consequences. DARU J Pharm Sci 22, 53. 10.1186/2008-2231-22-53 PMC4080727 24980396

[28] da Teixeira Silva JA and Nazarovets S (2025) The publish or perish, publish and perish, publish then perish, and now retract and perish cultures in academia. Naunyn Schmiedeberg's Arch Pharmacol 399, 3115–3131. 41071313 10.1007/s00210-025-04651-5

[29] Behar‐Horenstein LS , Roberts KW and Dix AC (2010) Mentoring undergraduate researchers: an exploratory study of students' and professors' perceptions. Mentoring & Tutoring 18, 269–291.

[30] Manathunga C (2007) Supervision as mentoring: the role of power and boundary crossing. Stud Contin Educ 29, 207–221.

[31] Gewin V (2012) Mental health: under a cloud. Nature 490, 299–301. 23066544 10.1038/nj7419-299a

[32] Levecque K , Anseel F , De Beuckelaer A , Van Der Heyden J and Gisle L (2017) Work organization and mental health problems in PhD students. Res Policy 46, 868–879.

[33] Evans T , Bira L , Gastelum J , Weiss LT and Vanderford NL (2018) Evidence for a mental health crisis in graduate education. Nat Biotechnol 36, 282–284. 29509732 10.1038/nbt.4089

[34] Woolston C (2019) PhDs: the tortuous truth. Nature 575, 403–406. 31723297 10.1038/d41586-019-03459-7

[35] Tenenbaum HR , Crosby FJ and Gliner MD (2001) Mentoring relationships in graduate school. J Vocat Behav 59, 326–341.

[36] Müller R and de Rijcke S (2017) Thinking with indicators. Exploring the epistemic impacts of academic performance indicators in the life sciences. Research Evaluation 26, 157–168.

[37] Fung FM , Blanc E and Coumoul X (2024) Digital futures of learning pharmacology and medicinal and organic chemistry. ACS Pharmacol Transl Sci 7, 1191–1194. 38633594 10.1021/acsptsci.4c00043 PMC11020058

[38] Fung FM (2024) Always by your side: a biochemical bonanza at the 48th FEBS congress in Milano. *FEBS Network*.

[39] Fung FM , Lam Y , Yap J , Musalli DA , Han JY , Togo K and Kim Y (2021) ChemPOV: digitizing an organic chemistry boardgame to support online learning. IEEE International Conference on Engineering, Technology & Education (TALE), 905–909.

[40] Kon MWR , Teo J , Fung FM and Potgieter M (2024) Two young observers at the WCC in the Hague share their reflections. Chem Int 46, 22–25.

[41] Teo J , Sharma P , Kon MWR , Han JY , Tan TLY , Teh YL , Yap JL and Fung FM (2025) Changing attitudes toward organic chemistry via a digital multiplayer game. J Chem Educ 102, 1476–1491.

[42] Sharma P , Teo J , Kon MWR , Han JY and Fung FM (2025) ChemPOV: evaluating a digital game‐based learning tool for organic chemistry through student‐researcher collaboration. Journal of Applied Learning & Teaching 8, 55–63.

[43] Ghosh K and Asyiqin D (2025) FEBS‐IUBMB‐ENABLE conference crosses seven seas: report on the 3(rd) International Molecular Biosciences PhD and Postdoc Conference – “Artificial intelligence: reshaping biomedical and healthcare research”. FEBS Open Bio 15, 3–13. 10.1002/2211-5463.70031 PMC12116934 40432230

[44] Hokanson SC , Grannan S , Greenler R , Gillian‐Daniel DL , Campa H and Goldberg BB (2019) A study of synchronous, online professional development workshops for graduate students and postdocs reveals the value of reflection and community building. Innov High Educ 44, 385–398.

[45] Li H , Öchsner A and Hall W (2017) Application of experiential learning to improve student engagement and experience in a mechanical engineering course. Eur J Eng Educ 44, 283–293.

[46] Machost H and Stains M (2023) Reflective practices in education: a primer for practitioners. CBE Life Sci Educ 22, Es2. 36972335 10.1187/cbe.22-07-0148 PMC10228263

[47] Káplár‐Kodácsy K and Dorner H (2020) The use of audio diaries to support reflective mentoring practice in Hungarian teacher training. Int J Mentor Coach Educ 9, 257–277.

[48] Hubbs DL and Brand CF (2005) The paper mirror: understanding reflective journaling. J Exp Educ 28, 60–71.

[49] Epp S (2008) The value of reflective journaling in undergraduate nursing education: a literature review. Int J Nurs Stud 45, 1379–1388. 18325522 10.1016/j.ijnurstu.2008.01.006

[50] Colomer J , Serra T , Cañabate D and Bubnys R (2020) Reflective learning in higher education: active methodologies for transformative practices. Sustainability 12, 3827.

[51] Lubbe W and Botha CS (2020) The dimensions of reflective practice: a teacher educator's and nurse educator's perspective. Reflective Pract 21, 287–300.

[52] Lasater K and Nielsen A (2009) Reflective journaling for clinical judgment development and evaluation. J Nurs Educ 48, 40–44. 19227755 10.3928/01484834-20090101-06

[53] Lama A (2023) Reflective teaching journals as an effective embedded formative assessment process of teaching skill development confidence in a longitudinal medical student‐as‐teacher elective. Medical Science Educator 33, 1493–1503. 38188416 10.1007/s40670-023-01938-1 PMC10766913

[54] Byars‐Winston AM , Branchaw J , Pfund C , Leverett P and Newton J (2015) Culturally diverse undergraduate researchers' academic outcomes and perceptions of their research mentoring relationships. Int J Sci Educ 37, 2533–2554. 27065568 10.1080/09500693.2015.1085133 PMC4822509

